# Hippocampal-Avoidance Whole-Brain Radiotherapy: Dosimetric Comparison of 3D-CRT, IMRT, and VMAT for Brain Metastases from Lung Cancer

**DOI:** 10.3390/cancers17233744

**Published:** 2025-11-24

**Authors:** Le Ba Thach, Mai Thi Thao, Nguyen Viet Nghia, Tran Nhat My, Duong Thanh Tai, Nissren Tamam, Abdelmoneim Sulieman, Hiba Omer, Hind Toufig, David Bradley

**Affiliations:** 1 Department of Radiation Oncology, Phuc Thinh General Hospital, Thanh Hoa 440000, Vietnam; lebathach1997@gmail.com (L.B.T.); maithao940515@gmail.com (M.T.T.); vietnghia285@gmail.com (N.V.N.); dr.trannhatmy@gmail.com (T.N.M.); 2 Department of Medical Physics, Faculty of Medicine, Nguyen Tat Thanh University, 298-300A Nguyen Tat Thanh Street, Ward 13, District 4, Ho Chi Minh City 700000, Vietnam; 3 Department of Physics, College of Science, Princess Nourah Bint Abdulrahman University, P.O Box 84428, Riyadh 11671, Saudi Arabia; nmtamam@pnu.edu.sa; 4 Biomedical Physics Department, Research and Innovation, King Faisal Specialist Hospital and Research Center, Riyadh 11211, Saudi Arabia; mohamedab@ksau-hs.edu.sa; 5 Department of Radiological Sciences, College of Applied Medical Sciences, King Saud Bin Abdulaziz University for Health Sciences, Riyadh 11481, Saudi Arabia; 6 Department of Radiological Sciences, College of Applied Medical Sciences, Imam Abdulrahman Bin Faisal University, P. O. Box 1982, Dammam 34212, Saudi Arabia; hbomer@iau.edu.sa; 7 Department of Surgery, Division of Radiology, College of Medicine, King Faisal University, Hofouf 31982, Saudi Arabia; htoufig@kfu.edu.sa; 8 Applied Radiation Physics and Technologies Group, CCDCU, Sunway University, Petaling Jaya 46150, Malaysia; d.a.bradley@surrey.ac.uk; 9 School of Mathematics and Physics, University of Surrey, Guildford GU2 7XH, UK

**Keywords:** brain metastases, whole-brain radiotherapy, hippocampal-avoidance WBRT, WBRT, VMAT, IMRT, 3D-CRT, HS-WBRT, HA-WBRT

## Abstract

**Simple Summary:**

Hippocampal-avoidance whole-brain radiotherapy (HA-WBRT) has become an important strategy for reducing neurocognitive decline in patients receiving whole-brain radiotherapy for brain metastases. However, the implementation of HA-WBRT varies widely depending on the technology available at each treatment center. In many low- and middle-income settings, advanced techniques such as volumetric-modulated arc therapy (VMAT) may not be accessible, making it essential to evaluate whether simpler techniques can offer comparable benefits. This study compares 3D-CRT, IMRT, and VMAT techniques for HA-WBRT in patients with lung cancer brain metastases. Fifteen treatment plans were created and evaluated for target dose coverage and organ-at-risk protection. Both IMRT and VMAT substantially reduced hippocampal dose compared with 3D-CRT. VMAT provided faster treatment delivery, while IMRT achieved similar dosimetric results using simpler and more accessible technology. Our findings support IMRT as a practical and feasible approach for delivering HA-WBRT in resource-limited environments, helping expand access to this important neuroprotective technique.

**Abstract:**

Background: This study was designed as a dosimetric feasibility analysis to compare hippocampal-avoidance whole-brain radiotherapy (HA-WBRT) using 3D-CRT, IMRT, and VMAT techniques, with particular attention to clinical applicability in resource-limited settings. While 3D-CRT was used as a reference for conventional WBRT, the primary aim was to determine whether IMRT can serve as an effective and accessible alternative to VMAT for HA-WBRT in centers without advanced technology infrastructure. Methods: Fifteen patients undergoing WBRT for symptom relief were planned using 3D-CRT, IMRT, and VMAT on the Elekta Monaco 6.1.4.0 system. Key organs at risk (OARs) such as the optic nerves, chiasm, eyes, and lenses were considered in the treatment planning. Plans were evaluated based on PTV dose distribution, Conformity Index (CI), Homogeneity Index (HI), and OAR dose constraints (RTOG 0933, NRG-CC001). Gamma pass rate analysis (3%/3 mm) was performed for the IMRT and VMAT plans. Results: IMRT and VMAT significantly reduced the hippocampal dose compared to 3D-CRT, with similar PTV coverage and OAR sparing. The mean D_max_ for the hippocampus was 15.4 Gy for IMRT and 15.5 Gy for VMAT compared to 31.2 Gy for 3D-CRT. The D100% for the hippocampus was 7.5 Gy for IMRT and 7.6 Gy for VMAT, both well below the RTOG 0933 threshold of 9 Gy, while 3D-CRT delivered 30.3 Gy. Additionally, IMRT and VMAT delivered lower doses to the optic nerves and chiasm. QA results showed gamma pass rates above 96% for all plans. This study focused solely on treatment-planning and dosimetric feasibility without evaluating patient outcomes or clinical follow-up. Conclusions: HA-WBRT with IMRT and VMAT significantly reduced the hippocampal dose while maintaining optimal PTV coverage. VMAT is preferred for its balance of efficacy, protection, and treatment time, while IMRT represents a feasible approach for facilities without VMAT, though it requires stricter dose control and longer treatment times.

## 1. Introduction

Brain metastases, particularly from lung cancer, represent a significant clinical challenge. Whole-brain radiotherapy (WBRT) remains a cornerstone of treatment for patients with multiple brain metastases, offering effective intracranial tumor control [1]. Hippocampal-sparing whole-brain radiotherapy (HS-WBRT) or hippocampal avoidance in whole-brain radiation therapy (HA-WBRT) has emerged as an effective strategy to mitigate cognitive decline associated with traditional WBRT in patients with brain metastases [2]. The hippocampus, specifically the sub-granular zone of the dentate gyrus, is crucial for memory formation, and its radiation-induced injury has been implicated in early cognitive deterioration following WBRT [3]. Studies have demonstrated that even low doses of radiation to the hippocampus can disrupt neurogenesis and impair memory recall [4]. HA-WBRT techniques address this, aiming to deliver radiation to the brain while sparing the hippocampal neural stem-cell niche, thus preserving cognitive function [5]. Clinical trials, such as RTOG 0933, have shown that HA-WBRT can significantly reduce hippocampal radiation exposure by up to 80%, leading to better memory preservation and quality of life compared to conventional WBRT [2]. Combining HA-WBRT with neuroprotective agents like memantine has further improved cognitive outcomes in patients [6]. A phase III trial (NRG Oncology CC001) is ongoing to validate these findings and explore the impact of HA-WBRT in combination with memantine on cognitive function in brain metastasis patients [7]. Early results suggest that this approach not only preserves memory but also provides adequate control of brain metastases, making HA-WBRT a promising advancement in neuroprotective radiation therapy [8].

The development of conformal radiation techniques has made HA-WBRT practically feasible. Three-dimensional conformal radiotherapy (3D-CRT) is a basic technique available in most radiation centers; however, its geometric limitations often make it suboptimal for sparing small, critical structures like the hippocampus. Intensity-modulated radiotherapy (IMRT) and volumetric modulated arc therapy (VMAT) are more advanced techniques that allow for improved dose conformity and sparing of organs at risk (OARs), particularly the hippocampi. Among these, VMAT has become the most widely used and preferred technique for HA-WBRT in many clinical settings. Its ability to deliver radiation in a continuous arc allows for improved dose distribution, offering better sparing of the hippocampus and other organs at risk (OARs) and shorter treatment times. Recent dosimetric studies and clinical planning investigations have consistently demonstrated VMAT’s advantages in target coverage and dose homogeneity for HA-WBRT, supporting its increasing adoption worldwide [9,10,11].

However, access to advanced radiotherapy technologies like VMAT remains limited in many low- and middle-income countries including Vietnam. In several hospitals across Vietnam, linear accelerators (LINACs) may only be equipped for IMRT, with no VMAT capabilities available [12,13,14]. While IMRT is more accessible and technically feasible in such settings, its effectiveness and practicality in delivering HA-WBRT—compared to VMAT—need further evaluation.

Therefore, this study was designed as a dosimetric feasibility analysis comparing 3D-CRT, IMRT, and VMAT for hippocampal-avoidance whole-brain radiotherapy (HA-WBRT). It focuses exclusively on treatment planning and dosimetric performance, rather than clinical outcomes. A key objective is to assess the feasibility and clinical relevance of using IMRT for HA-WBRT in resource-constrained environments like Vietnam, where VMAT may not be an available option. By identifying whether IMRT can provide acceptable hippocampal sparing and overall treatment quality, this study could help broaden access to neurocognitive-preserving radiotherapy for brain metastasis patients in settings with limited technology. Unlike previous studies that assume access to advanced radiotherapy systems, our study is distinct in assessing whether IMRT, which is more widely available in lower-resource hospitals, can deliver clinically acceptable HA-WBRT. This aspect is especially relevant for expanding hippocampal-sparing radiotherapy to under-served populations.

## 2. Materials and Methods

### 2.1. Patients

Over the period June 2024 and August 2025, a group of 15 patients with brain metastases from lung cancer were treated at Phuc Thinh General Hospital, each receiving palliative whole-brain radiotherapy (WBRT). The group included two patients diagnosed with small-cell lung cancer (SCLC) and eight with non-small-cell lung cancer (NSCLC). All 15 patients were in good general condition at the time of treatment, alert, and compliant with medical instructions.

### 2.2. Equipment

A comprehensive treatment process was implemented, from data collection to treatment delivery. CT images were acquired using a Discovery RT32 slice scanner (GE HealthCare, Chicago, IL, USA). For radiation therapy planning and contouring, the CT data were transferred automatically to Monaco Planning Software (version 6.1.4.0).

For quality assurance (QA), both IMRT and VMAT plans were evaluated using the Matrix Resolution System and MyQA Accept Software (2024-001) (IBA), ensuring that these met the accuracy and safety standards before patient treatment.

The radiation therapy was delivered using the Elekta Synergy Linear Accelerator (LINAC) (Elekta Oncology Systems, Crawley, UK) equipped with an Agility head and a 160 multi-leaf collimator (MLC). Each leaf measures 0.5 cm in width and moves at a speed of 3.5 cm/s. The system supports both 6 MV and 10 MV photon beams, enhancing precision, optimizing dose delivery, and reducing treatment time while improving normal tissue sparing during IMRT and VMAT radiotherapy.

### 2.3. Methodology

#### 2.3.1. Patient Simulation CT Imaging and Volume Delineation Data

In each case, the patient was positioned supine and immobilized following the technician’s guidance. A three-point mask was used to stabilize the head, the laser center being aligned and marked as the simulation center. A simulation CT scan was performed using the GE Discovery RT 32-slice scanner (GE HealthCare, Chicago, IL, USA) with a slice thickness of 2.5 mm. Although the RTOG 0933 protocol recommends a 1.25 mm slice thickness, this was not achievable due to equipment limitations. Moreover, MRI fusion was not performed, acknowledged to be a limitation.

The CT data were analyzed by physicians to delineate the Planning Target Volume (PTV) and Organs at Risk (OARs) [7]. Hippocampal contours were delineated in accordance with the RTOG 0933 atlas by experienced radiation oncologists. Observed were the follows:

- CTV (Clinical Target Volume): This covers the entire brain up to C1 if there is no posterior fossa metastasis, or up to C2 if posterior fossa metastasis is present.
- PTV (Planning Target Volume): This is defined as the CTV + 3 mm, excluding the hippocampal margin.
- OARs (Organs at Risk): These include the eyeballs, lenses, optic nerves (left and right), optic chiasm, hippocampus, and hippocampal avoidance zone (hippocampus + 5 mm margin).

**Figure 1:**
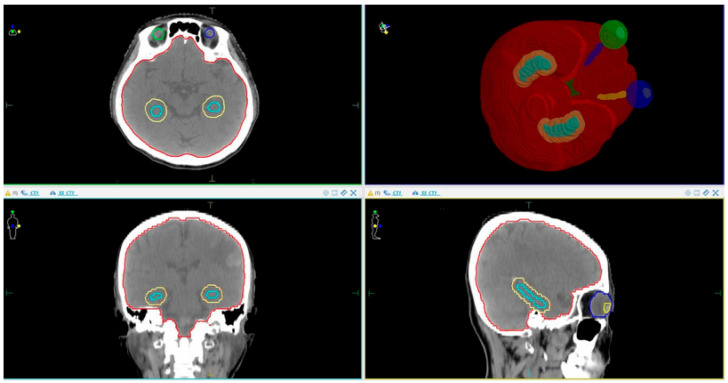
PTV +5 mm and OARs location. The red line represents the PTV, the yellow line represents the hippocampus +5 mm margin, while the remaining structures indicate the eyes and optic nerves.

The anatomical relationship between the PTV and surrounding OARs, including the hippocampus and avoidance zone, is illustrated in Figure 1. This visual reference helps clarify the spatial planning constraints and margin definitions used in this study.

#### 2.3.2. Radiotherapy Dose Prescription

According to RTOG 0933, the total dose for WBRT is 30 Gy, delivered in 10 fractions. The evaluation criteria are outlined in Table 1 and Table 2.

#### 2.3.3. Treatment Planning

**Figure 2:**
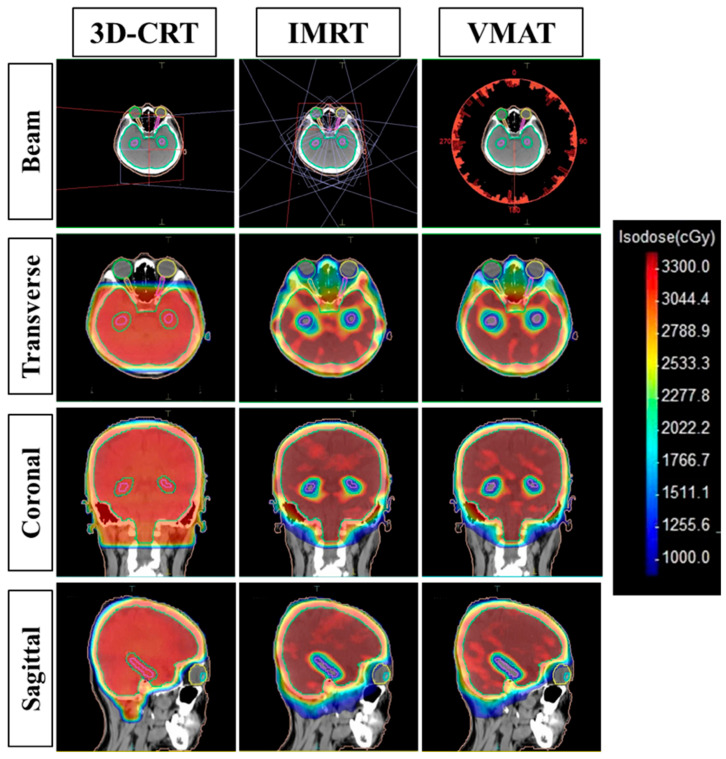
Dose distribution for Hippocampal-Avoidance Whole-Brain Radiotherapy using 3D-CRT, IMRT, and VMAT techniques.

Each patient underwent treatment planning using three different techniques (Figure 2): 3D-CRT, IMRT, and VMAT, all conducted on the Monaco software (version 6.2.2).

- 3D-CRT Planning: Designed with two opposed lateral fields at gantry angles of 90° and 270°, with a collimator rotation of 5° to optimize eye shielding. The field size was extended to cover the entire PTV. The Collapsed Cone algorithm was used for dose calculation.
- HA-WBRT using IMRT: This plan utilized nine fields, each with a collimator angle of 45°. In our study, the 45° collimator angle was selected because this practice is consistent with the literature [15,16], helping to reduce the tongue-and-groove effect and facilitating dose modulation in concave anatomical regions such as the hippocampus. The Monte Carlo algorithm was applied.
- HA-WBRT using VMAT: The plan incorporated a single beam consisting of three full arcs (360° rotation), the collimator being set to 45°.

#### 2.3.4. Treatment Plan Evaluation Criteria

In addition to tumor coverage percentage, the homogeneity index (HI) and conformity index (CI) are two other critical parameters for evaluating the effectiveness of a treatment plan in tumor management [17,18,19].

Homogeneity Index:

(1) $$\mathrm{HI}\;=\;\frac{D2\%-D98\%}{Drx}\times 100\%$$

where:

- D2% is the absorbed dose value close to the D_max_, received by 2% of the PTV volume;
- D98% is the absorbed dose value close to the D_min_, received by 98% of the PTV volume;
- Drx is the prescribed dose to the PTV volume.

The HI approaching 0 indicates a uniform dose distribution within the target volume. The closer HI is to 0, the better the radiotherapy quality.

The Conformity Index indicates the ratio between the volume covered by the reference isodose line (typically 95% according to ICRU) and the prescribed PTV volume.

(2) $$\mathrm{CI}=\frac{Vri}{TV}$$

where:

- Vri is the volume enclosed by the reference isodose line;
- TV is the PTV volume.

The CI has an ideal value of 1, with values closer to 1 indicating higher dose conformity to the designated target volume [20].

#### 2.3.5. Treatment Plan Quality Assurance (QA Plan)

**Figure 3:**
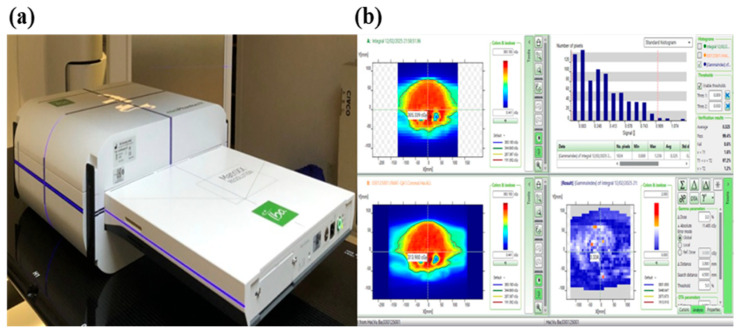
QA equipment. (**a**) Matrixx Resolution and Mini R phantom; (**b**) MyQA software.

The equipment utilized in implementing the QA plan is illustrated in Figure 3, featuring the Matrixx Resolution system, Mini R phantom, and MyQA software (2024-001). The Matrixx Resolution system is equipped with 1521 ionization chambers arranged in a 39 × 39 matrix, allowing for highly detailed dose distribution analysis while minimizing interpolation errors. With a detector spacing of just 6.5 mm (center-to-center), this high-resolution setup ensures precise measurements, especially for advanced techniques like VMAT, which involve steep dose gradients. Its expansive 25.3 × 25.3 cm^2^ active measurement area enables efficient verification of large treatment fields.

The dose distribution of the Monaco-calculated plan was compared to the actual radiation dose using the gamma index method, with results expressed as the gamma pass rate [21].

#### 2.3.6. Statistical Analysis

Statistical analyses were performed using GraphPad Prism (version 9.0). The GraphPad *t*-test module was configured with the following settings: paired design, two-tailed hypothesis testing, assumed Gaussian distribution, 95% confidence intervals. Three pairwise comparisons were conducted for each dosimetric parameter: (p1) 3D-CRT vs. IMRT, (p2) 3D-CRT vs. VMAT, and (p3) IMRT vs. VMAT. A statistically significant difference was defined as *p* < 0.05.

## 3. Results

### 3.1. QA Plan

**Figure 4:**
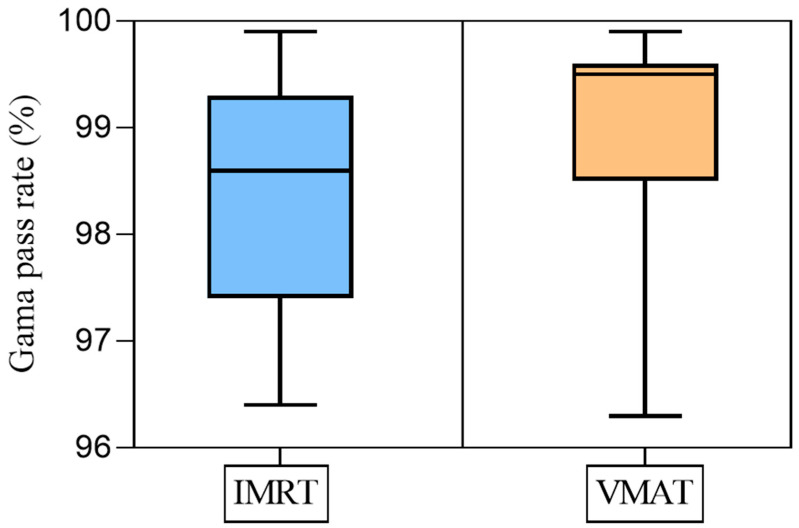
Gamma pass rate for the IMRT and VMAT plans.

The gamma analysis criterion of 3%/3 mm was used for evaluation. Figure 4 displays the distribution of the gamma pass rate values for both the IMRT and VMAT techniques.

It is clear that the VMAT radiation plans exhibited higher gamma pass rates than IMRT, with 93% of VMAT plans exceeding a pass rate of 98% compared to 73% for IMRT. However, all plans had a gamma pass rate greater than 96%, meeting the requirement for clinical treatment [22].

### 3.2. Dosimetric Evaluation

The findings of the analysis on tumor coverage efficiency for 3D-CRT, VMAT, and IMRT are presented in Table 3 and Table 4. It is evident that 3D-CRT demonstrated superior tumor coverage efficiency, with a D98% value of 29.3 ± 0.5 Gy, exceeding that of both VMAT and IMRT by 13.6% and 16.7%, respectively. The V30Gy for both 3D-CRT and VMAT exceeded 95%, whereas for IMRT, it was 93.9%. In terms of hotspot control, 3D-CRT achieved the lowest D2% value (32.0 ± 0.4 Gy), while the D2% values for VMAT and IMRT, although greater than 35 Gy, remained significantly below the allowable limits set forth in the RTOG 0933 and NRG CC001 guidelines.

The absence of significant differences in the Homogeneity Index (HI) and Conformity Index (CI) between VMAT and IMRT, as shown in Table 4, suggests that both techniques offer comparable dose uniformity and coverage within the tumor region. Additionally, the lower HI value observed for 3D-CRT supports its superior dose uniformity in the Planning Target Volume (PTV).

**Figure 5:**
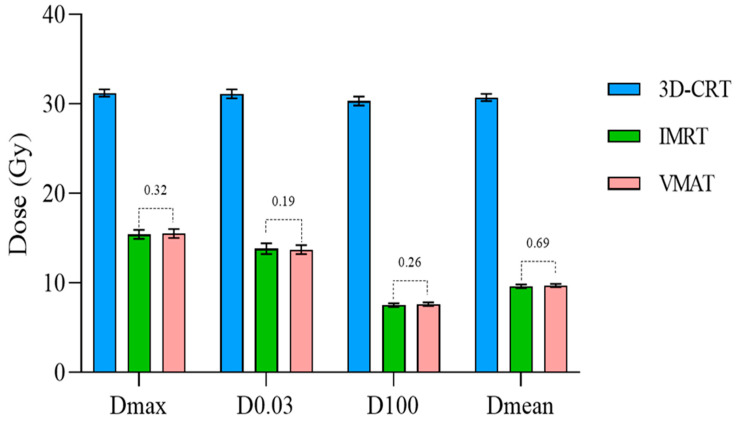
Dose distribution to the hippocampus.

Table 5 and Figure 5 highlight the superiority of IMRT and Volumetric Modulated Arc Therapy (VMAT) in minimizing radiation exposure to healthy organs during radiotherapy. With IMRT, the hippocampus exhibited the lowest D100% and D_mean_ values, measuring 7.5 ± 0.2 Gy and 9.6 ± 0.2 Gy, respectively. VMAT plans produced nearly identical values, with D100 at 7.6 ± 0.2 Gy and D_mean_ at 9.7 ± 0.2 Gy, both well below the prescribed limits. In contrast, 3D-CRT resulted in significantly higher values for the hippocampus, with D100 at 30.3 ± 0.5 Gy and D_mean_ at 30.7 ± 0.4 Gy, which are nearly four times greater than the recommended limit for hippocampal protection. Regarding the hotspot area within the hippocampus, D_max_ and D100% values for both IMRT and VMAT were similar, ranging from 12.9 Gy to 16.0 Gy, not exceeding the required dose limits of 16 Gy.

For the chiasm and both the left and right optic nerves, the lowest D_max_ values were observed with IMRT, at 29.0 Gy, 27.8 Gy, and 27.7 Gy, respectively. VMAT produced slightly greater values, though the difference was not statistically significant. The greatest Dmax values were seen with the 3D-CRT technique, with all values exceeding 30 Gy. Although 3D-CRT resulted in the lowest dose indices for the lenses (L/R) and eyes (L/R), the doses from both VMAT and IMRT were still sufficiently low, meeting all the proposed safety criteria.

**Figure 6:**
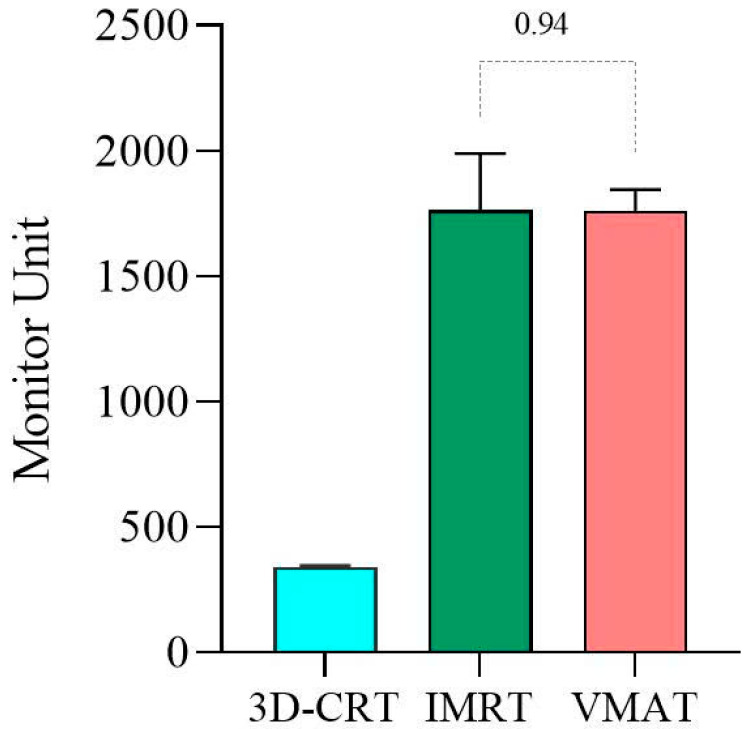
Average MU value for 3D-CRT, IMRT, and VMAT.

Figure 6 illustrates the calculated MU for treatment plans using 3D-CRT, IMRT, and VMAT. 3D-CRT, possessing the least sophisticated dose modulation capabilities, exhibited the lowest mean MU (340). In contrast, IMRT had the highest average MU at 1764, while VMAT had a slightly lower average of 1761. As shown in Figure 6, 3D-CRT exhibited the lowest mean MU (340), due in part to its simplicity. Conversely, IMRT required the highest mean MU (1764) as a result of the use of multiple fixed fields. The VMAT technique resulted in a mean treatment time of approximately 6 min, nearly half the 12 min required for IMRT. This finding supports the existing literature indicating that VMAT reduces both MU and delivery time, thereby improving clinical workflow and patient throughput.

## 4. Discussion

This study demonstrated the superiority of IMRT and VMAT in preserving the hippocampus during whole-brain radiotherapy. Specifically, in the hippocampus region, the D_max_ was reduced by approximately 50.6%, D0.03 cm^3^ by 49.5%, D100% by 75.2%, and the D_mean_ by 68.7% when using VMAT and IMRT compared to the 3D-CRT technique. Notably, all treatment plans adhered to the criteria outlined in the RTOG 0933 guidelines, effectively mitigating the risk of radiation-induced damage to brain regions associated with memory function.

The findings of this study align closely with previous research on hippocampal-avoidance radiotherapy [15,23,24,25]. Mathew et al. [26] reported outcomes from a group of 12 patients treated with hippocampal-avoidance whole-brain radiotherapy using the VMAT technique on the Elekta VersaHD system, achieving a median PTV V30 Gy of 96.53% and a median D98% of 28.27 Gy. In comparison, our study showed slightly lower values of 95.3% and 25.8 Gy, respectively. For the hippocampus, Mathew’s D100% values were 8.76 Gy and 8.86 Gy for the left and right sides, respectively, while our results were notably lower, with a mean value of 7.6 Gy across the entire region. Unfortunately, no data on the sparing of other organs at risk (OARs) were provided in that study, limiting further comparison. Alexander et al. [15], using IMRT, reported a PTV V30Gy of 92% and a D98% of 25.37 Gy. Our corresponding results were 93.9% and 25.1 Gy, respectively. Although the D98% of the IMRT plans (25.1 Gy) was lower than that of VMAT (25.8 Gy), it still met the minimum requirement of ≥22.5 Gy as defined by NRG-CC001, thereby confirming its clinical acceptability. For hippocampal dose, our mean D100% was 7.5 Gy—significantly lower than the 8.37 Gy reported by Alexander—underscoring our focus on minimizing hippocampal exposure while maintaining clinically acceptable target coverage. Both Mathew and Alexander incorporated treatment table rotation to optimize plan quality. In contrast, our approach maintained a fixed table position throughout planning and delivery. While rotation can enhance hippocampal sparing, particularly in IMRT, it may extend treatment times and introduce potential setup uncertainties due to manual adjustments. To mitigate these concerns, we used a consistent 45° collimator rotation across all beam angles, which reduced the tongue-and-groove effect, improved dose distribution uniformity, and minimized dose variation—without compromising workflow efficiency [27].

Specifically, an additional focus of this study is the clinical applicability of IMRT in hippocampal-protective whole-brain radiotherapy. As illustrated in Table 3, IMRT demonstrated a 1.1% higher D2%, a 2.7% lower D98%, and a 1.5% lower V30Gy compared to VMAT. While IMRT showed a slight disadvantage in tumor coverage relative to VMAT, the observed differences remained within acceptable clinical thresholds. Furthermore, an assessment of dosimetric parameters for adjacent healthy organs (Table 5) revealed minimal differences between IMRT and VMAT, with IMRT exhibiting a slight advantage in dose reduction to the hippocampus, optic nerves, and the right eye. Overall, IMRT demonstrated satisfactory tumor coverage and organ-at-risk protection, supporting its clinical utility.

Nevertheless, a comprehensive evaluation of IMRT feasibility must consider factors such as monitor units (MU) and treatment duration. In this study, the total MU for IMRT was 1764—comparable to VMAT—with nine treatment fields and an average treatment duration of 12 min, which is consistent with standard practice for head and neck radiotherapy cases [28,29]. In addition to comparable dosimetric outcomes, VMAT demonstrated clear advantages in treatment efficiency. The reduced MU and shorter beam-on time observed with VMAT (approximately 6 min) compared to IMRT (approximately 12 min) can significantly enhance clinical throughput and patient comfort. This is particularly valuable in high-volume or resource-constrained settings, where treatment time impacts machine availability and patient scheduling. However, longer treatment delivery in IMRT may cause patient discomfort, especially for elderly or debilitated patients who need to maintain a stable position for a longer period. In busy departments, this may also reduce patient throughput and increase daily machine workload.

While the dosimetric and technical findings of this study are encouraging, translating these results into routine clinical practice can be challenging, especially in centers with limited technological and human resources. In many developing regions, the availability of advanced treatment planning systems, QA infrastructure, and adequately trained personnel remains a significant constraint. Therefore, beyond the dosimetric comparison, it is essential to consider the practical aspects of implementing hippocampal-avoidance IMRT under such conditions. Implementing IMRT for hippocampal-avoidance WBRT in resource-limited settings may face several challenges including limited access to advanced treatment planning systems, reduced machine availability, and insufficient staff training in complex plan optimization and QA. To overcome these barriers, centers can adopt standardized beam templates, simplify QA workflows, and conduct targeted training programs in collaboration with experienced institutions. Establishing cooperative networks and sharing planning resources across institutions can further enhance treatment quality and ensure the safe, efficient use of IMRT where VMAT capability is not available.

Despite these encouraging findings, this study has several limitations that should be acknowledged. First, this study represents a pilot single-center dosimetric feasibility analysis with a limited sample size (*n* = 15), however, the cohort size remains modest. This is consistent with previous dosimetric investigations by Gondi et al. (five patients) [24], Hsu et al. (ten patients) [30], Nevelsky et al. (ten patients) [15], and Prokic et al. (ten patients) [31], which similarly focused on treatment planning optimization rather than clinical outcomes. Future work will incorporate a larger patient cohort and power analysis to improve statistical robustness and generalizability. Second, MRI-based hippocampal contouring was not incorporated due to the lack of MRI fusion capability at the time of treatment planning. Future studies will employ CT images with 1.25 mm slice thickness and MRI co-registration following RTOG 0933 recommendations to improve anatomical accuracy. Third, the current analysis focused exclusively on dosimetric and planning parameters without including patient outcome data or neurocognitive assessments. Future work will extend these findings to prospective clinical validation correlating hippocampal dose metrics with memory and cognitive outcomes. Finally, implementing HA-WBRT using IMRT in resource-limited centers remains challenging due to machine availability, staff training, and QA capacity. However, strategies such as standardized beam templates, simplified QA workflows, and collaborative training initiatives could help overcome these barriers and make hippocampal-avoidance radiotherapy more accessible worldwide.

## 5. Conclusions

This study further supports the superiority of IMRT and VMAT in HA-WBRT. All PTV and OAR parameters for IMRT and VMAT plans met the RTOG 0933 criteria, effectively minimizing the risk of cognitive impairment while preserving therapeutic efficacy. IMRT also demonstrated feasibility in achieving adequate tumor coverage alongside hippocampal and OAR sparing, making it a viable option for centers without access to VMAT technology.

## Figures and Tables

**Table 1:** RTOG 0933 Guidelines for WBRT.

Parameter	Per Protocol	Variation Acceptable
PTV	D2% ≤ 37.5 Gy	D2% > 37.5 Gy, ≤ 40 Gy
	D98% ≥ 25 Gy	D98% < 25 Gy
Hippocampus	D100% ≤ 9 Gy	D100% ≤ 10 Gy
	Maximum dose ≤ 16 Gy	Maximum dose ≤ 17 Gy
Optic Nerves and Chiasm	Maximum dose ≤ 37.5 Gy	Maximum dose ≤ 37.5 Gy

**Table 2:** NRG CC001 Guidelines for WBRT.

Structure	NRG-CC001 Dose Constraints	NRG-CC001 Dose Constraints–Variation Acceptable
PTV	D2% ≤ 37.5 Gy	D2% ≤ 37.5 to 40 Gy
	D98% ≥ 25 Gy	D98% 22.5 to 25.0 Gy
	V30Gy ≥ 95%	V30Gy 90 to 95%
Hippocampus, Right	D100% ≤ 9 Gy	D100% ≤ 9 Gy to 10 Gy
	D0.03cc ≤ 16 Gy	D0.03cc ≤ 16 to 17 Gy
Hippocampus, Left	D100% ≤ 9 Gy	D100% ≤ 9 Gy to 10 Gy
	D0.03cc ≤ 16 Gy	D0.03cc ≤ 16 to 17 Gy
Optic Nerve, Right	D0.03cc ≤ 30 Gy	D0.03cc ≤ 30 to 37.5 Gy
Optic Nerve, Left	D0.03cc ≤ 30 Gy	D0.03cc ≤ 30 to 37.5 Gy
Optic Chiasm	D0.03cc ≤ 30 Gy	D0.03cc ≤ 30 to 37.5 Gy

**Table 3:** Dose delivery to PTV.

Dose Index	3D-CRT	IMRT	VMAT	p1	p2	p3
D2% (Gy)	32.0 ± 0.4	35.5 ± 0.4	35.1 ± 0.3	<0.0001	<0.0001	0.0072
D98% (Gy)	29.3 ± 0.5	25.1 ± 0.7	25.8 ± 0.6	<0.0001	<0.0001	0.0014
V30Gy (%)	95.5 ± 1.5	93.9 ± 0.6	95.3 ± 0.6	0.0007	0.5209	<0.0001

**Table 4:** CI and HI evaluation.

	3D-CRT	IMRT	VMAT	p1	p2	p3
HI	0.11 ± 0.06	0.35 ± 0.02	0.31 ± 0.02	<0.0001	<0.0001	0.0011
CI	0.98 ± 0.02	0.96 ± 0.01	0.96 ± 0.01	0.0007	<0.0001	0.8275

**Table 5:** Dose distribution to OARs.

OARs	Dose Index	3D-CRT	IMRT	VMAT	p1	p2	p3
Hippocampus	D_max_ (Gy)	31.2 ± 0.4	15.4 ± 0.5	15.5 ± 0.5	<0.0001	<0.0001	0.3200
	D0.03 cm^3^ (Gy)	31.1 ± 0.5	13.8 ± 0.6	13.7 ± 0.5	<0.0001	<0.0001	0.1875
	D100% (Gy)	30.3 ± 0.5	7.5 ± 0.2	7.6 ± 0.2	<0.0001	<0.0001	0.2654
	D_mean_ (Gy)	30.7 ± 0.4	9.6 ± 0.2	9.7 ± 0.2	<0.0001	<0.0001	0.6927
Chiasm	D_max_ (Gy)	30.8 ± 0.5	29.0 ± 0.5	29.0 ± 0.8	<0.0001	<0.0001	0.6852
	D0.03 cm^3^ (Gy)	30.8 ± 0.4	28.7 ± 0.3	28.0 ± 1.2	<0.0001	<0.0001	0.0531
Left Lens	D_max_ (Gy)	2.7 ± 0.4	4.6 ± 0.2	4.7 ± 0.1	<0.0001	<0.0001	0.2843
Right Lens	D_max_ (Gy)	2.8 ± 0.5	4.6 ± 0.1	4.6 ± 0.1	<0.0001	<0.0001	0.6578
Left Optic Nerve	D_max_ (Gy)	30.8 ± 0.5	27.8 ± 1.3	28.2 ± 0.5	<0.0001	<0.0001	0.3341
	D0.03 cm^3^ (Gy)	30.5 ± 0.3	26.5 ± 1.5	26.9 ± 1.2	<0.0001	<0.0001	0.0349
Right Optic Nerve	D_max_ (Gy)	31.0 ± 0.3	27.7 ± 1.4	28.0 ± 1.3	<0.0001	<0.0001	0.3013
	D0.03 cm^3^ (Gy)	30.6 ± 0.4	26.2 ± 1.9	26.8 ± 1.6	<0.0001	<0.0001	0.0542
Left Eye	D_max_ (Gy)	11.2 ± 1.3	21.1 ± 1.0	21.1 ± 0.7	<0.0001	<0.0001	0.8741
Right Eye	D_max_ (Gy)	11.8 ± 1.9	20.6 ± 1.0	21.0 ± 1.1	<0.0001	<0.0001	0.2854

## Data Availability

Research data are stored in an institutional repository and will be shared upon request to the corresponding author.
